# Infection of B Cell Follicle-Resident Cells by Friend Retrovirus Occurs during Acute Infection and Is Maintained during Viral Persistence

**DOI:** 10.1128/mBio.00004-19

**Published:** 2019-02-19

**Authors:** Sonja Windmann, Lucas Otto, Camilla Patrizia Hrycak, Anna Malyshkina, Nadine Bongard, Paul David, Matthias Gunzer, Ulf Dittmer, Wibke Bayer

**Affiliations:** a Institute for Virology, University Hospital Essen, University Duisburg-Essen, Essen, Germany; b Institute for Experimental Immunology and Imaging, University Hospital Essen, University Duisburg-Essen, Essen, Germany; Columbia University; University of California, Irvine; Dartmouth Medical School

**Keywords:** follicular B cells, murine leukemia virus, persistence, retroviruses, viral immunity, viral pathogenesis

## Abstract

B cell follicles of the spleen and lymph nodes are immune privileged sites and serve as sanctuaries for infected CD4^+^ cells in HIV infection. It is assumed that CD8^+^ T cell responses promote the establishment of the reservoir, as B cell follicles do not permit CD8^+^ T cell entry. Here we analyzed the infected cell population in the Friend retrovirus (FV) infection and investigated whether FV can similarly infect follicular cells. For analysis of FV-infected cells, we constructed a recombinant FV encoding the bright fluorescent protein mWasabi and performed flow cytometry with cells isolated from spleens, lymph nodes and bone marrow of FV-mWasabi-infected mice. Using t-stochastic neighbor embedding for data exploration, we demonstrate how the target cell population changes during the course of infection. While FV was widely distributed in erythrocytes, myeloid cells, B cells, and CD4^+^ T cells in the acute phase of infection, the bulk viral load in the late phase was carried by macrophages and follicular B and CD4^+^ T cells, suggesting that FV persists in cells that are protected from CD8^+^ T cell killing. Importantly, seeding into follicular cells was equally observed in CD8^+^ T cell-depleted mice and in highly FV-susceptible mice that mount a very weak immune response, demonstrating that infection of follicular cells is not driven by immune pressure. Our data demonstrate that infection of cells in the B cell follicle is a characteristic of the FV infection, making this murine retrovirus an even more valuable model for development of retrovirus immunotherapy approaches.

## Abstract (precis)

Human immunodeficiency virus is notorious for its ability to avoid clearance by therapeutic interventions, which is partly attributed to the establishment of reservoirs in latently infected cells and cells that reside in immunologically privileged B cell follicles. In the work presented here, we show that cells of the B cell follicle are equally infected by a simple mouse gammaretrovirus. Using fluorescently labeled Friend retrovirus, we found that B cells and T cells in the B cell follicle, while not carrying the bulk of the virus load, were indeed infected by Friend virus in the early acute phase of the infection and persisted in the chronic infection. Our results suggest that infection of follicular cells may be a shared property of lymphotropic viruses and propose the FV infection of mice as a useful model to study strategies for follicular reservoir elimination.

## INTRODUCTION

In human immunodeficiency virus (HIV) infection, it has been recognized that a major hurdle for virus eradication is the establishment of a viral reservoir in privileged sites (1), which is similarly found in simian immunodeficiency virus (SIV)-infected macaques where it could be demonstrated that this reservoir is seeded very early in infection (2, 3). The reservoir is established in follicular CD4^+^ T cells that reside in B cell follicles of lymphoid tissues and are inaccessible for cytotoxic immune cells, as well as hard to reach by antiretroviral therapy, as therapeutic drug levels tend to be reduced in these tissues (4).

The Friend retrovirus (FV) is a murine gammaretrovirus complex consisting of Friend murine leukemia virus (F-MuLV) and spleen focus-forming virus (SFFV) (5) that causes lethal erythroleukemia and splenomegaly in genetically susceptible mice. In mice that are genetically resistant to FV-induced disease, FV establishes a chronic infection that is characterized by CD8^+^ T cell dysfunction that is mainly attributable to an expanded regulatory T cell (Treg) population (6, 7). Therapeutic interventions to eliminate persistent FV loads during the chronic phase of infection have been difficult to establish. Therapeutic depletion of Tregs resulted in a reduction of the viral load set point (8), as did the combination of Treg depletion with therapeutic administration of antibodies against inhibitory ligands on CD8^+^ T cells (9), but elimination of chronic virus was not achieved in the majority of chronically infected mice. Therapeutic immunizations of chronically infected mice have proven very difficult to establish and have been only mildly successful (10).

It has been described in the past that B cells constitute a major reservoir of infectious FV in the chronic phase of infection (11). Reasoning that either the location or identity of these B cells may be underlying the robustness of the chronic FV load, we aimed to identify the FV-infected B cell subsets. Therefore, we constructed an FV complex comprising a fluorescently labeled F-MuLV encoding the bright fluorescent protein mWasabi (12), which allows for easy detection of FV-infected cells, and performed advanced flow cytometric analysis of FV-mWasabi-infected cells, applying multiple staining panels to identify all cell types, as well as individual subpopulations, that are infected at different time points. Although limited by the fact that only F-MuLV-, but not SFFV-infected cells are readily detected after infection with our FV-mWasabi complex, our target cell analysis reveals a dynamic pattern of FV-mWasabi-infected cells over the course of infection and shows that follicular B and CD4^+^ T cells indeed contribute to the chronic FV load, although macrophages carry the bulk of chronic FV.

## RESULTS

### Construction of a bright fluorescently labeled FV.

**FIG 1:**
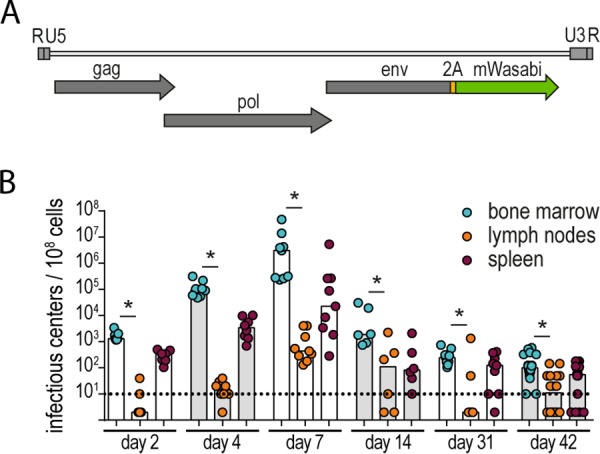
Construction of an F-MuLV encoding mWasabi. (A) For expression of mWasabi by F-MuLV, the mWasabi coding sequence was fused to the 3′ end of the envelope open reading frame, linked by a sequence encoding the self-cleaving 2A peptide from porcine teschovirus. FV-mWasabi was obtained after reconstitution of F-MuLV-mWasabi in complex with wild-type SFFV. (B) C57BL/6 mice were infected with 20,000 SFFU FV-mWasabi, and viral loads were determined at different time points after infection. Each circle represents the value for an individual mouse, and bars show median values of groups of mice. The dotted lines indicate the detection limit. The data for each time point were obtained from two (day 2, day 4, day 14, and day 31), three (day 7), or four (day 42) independent experiments (*n* = 7 [day 2], 8 [day 4], 9 [day 7], 6 [day 14], 9 [day 31], or 20 [day 42]). Statistically significant differences (*P < *0.05) between viral loads of the different organs per time point are indicated by short lines and asterisks.

The construction of a fluorescently labeled, replicating FV had been a difficult quest in the past; however, we were able to create an F-MuLV that encodes mWasabi fused to the envelope (Env) open reading frame. While direct fusion to the Env R peptide did allow for viral particle formation, the construct was very unstable, and fluorescence was lost after a few passages in cell culture. However, additional introduction of a self-cleaving 2A peptide from porcine teschovirus (Fig. 1A) (13) lent stability to the construct, and fluorescence of the recombinant virus was maintained for more than 15 passages *in vitro* (data not shown). After reconstitution of the FV complex comprising F-MuLV-mWasabi and wild-type SFFV, we infected C57BL/6 mice and isolated bone marrow, lymph nodes, and spleens at different time points. Analysis of the viral loads by conventional immunocytochemistry-based focal infectivity assay (14) confirmed that the replication kinetics of the mWasabi-labeled FV was unimpaired and indeed comparable to that of wild-type FV (15), with the highest virus loads observed in bone marrow and spleen samples at day 7 and low but stable virus loads in the late phase of infection (Fig. 1B). Of note, none of the mice were able to completely clear the infection, as we detected virus in all bone marrow samples on day 42, but the viral loads in the lymph nodes of half of the mice were below the detection limit at this time point, and again half of these mice also had undetectable viral loads in spleens.

### Dynamic pattern of FV-mWasabi-infected cell populations over the course of infection.

**FIG 2:**
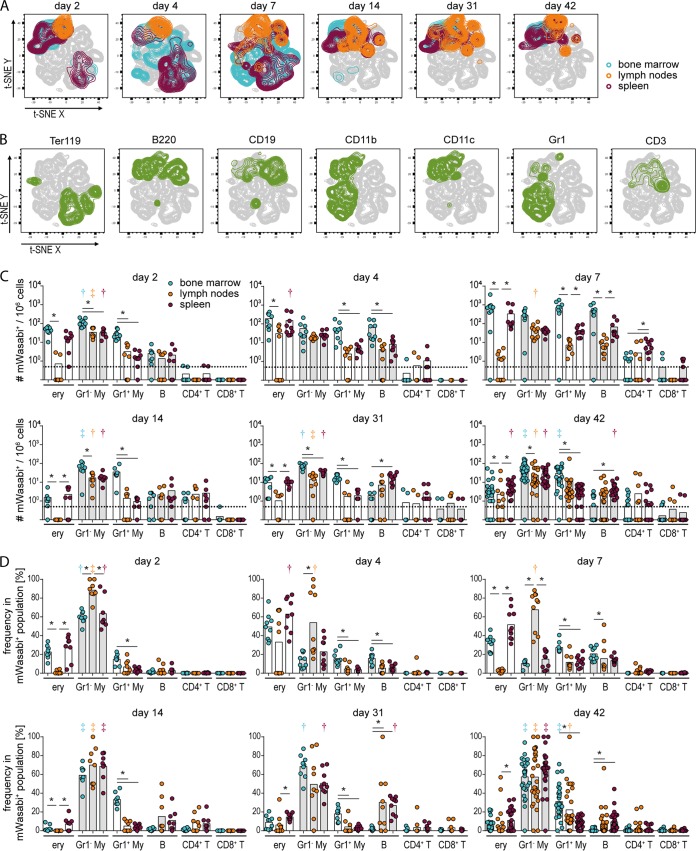
Analysis of FV-mWasabi-infected cells. C57BL/6 mice were infected with 20,000 SFFU FV-mWasabi, and infected target cells were analyzed in bone marrow, lymph nodes, and spleens on days 2, 4, 7, 14, 31, and 42 after FV-mWasabi infection. For the identification of infected cell types, isolated cells were stained with a target cell antibody panel and analyzed by flow cytometry. Minimal manual gating was performed to gate on singlets, live cells, and mWasabi^+^ cells, which were concatenated and subjected to a t-SNE analysis. (A) Manual gates on samples from individual organs and days of analysis were overlaid on the t-SNE plot to visualize the samples. (B) Manual gates on individual cell markers were overlaid on the t-SNE plot to identify cell types. (C and D) Manual gating was performed to determine the number of mWasabi^+^ cells in Ter119^+^ cells (ery), Gr1^−^ CD11b^+^ CD11c^+^ myeloid cells (Gr1^−^ My), Gr1^+^ CD11b^+^ myeloid cells (Gr1^+^ My), CD19^+^ B220^+^ B cells, and CD4^+^ or CD8^+^ CD3^+^ T cells, which is shown as the number of mWasabi^+^ cells in 10^6^ cells (C) and as the frequency of these cell types in the total population of mWasabi^+^ cells (D). Each circle represents the value for an individual mouse, and bars depict mean values for groups of mice. The dotted line indicates the detection limit. The data for each time point were obtained from two (day 2, day 4, day 14, and day 31), three (day 7), or six (day 42) independent experiments (*n* = 8 [day 2], 9 [day 4], 10 [day 7], 8 [day 14], 9 [day 31], or 28 [day 42]). Statistically significant differences (*P < *0.05) between the number (C) or frequency (D) of infected cells of the different organs within a cell subset are indicated by lines and asterisks; color-coded dagger or double-dagger symbols indicate dominant subsets with a significantly higher number (C) or frequency (D) of FV-mWasabi-infected cells compared to three (dagger) or four (double dagger) other subsets within one organ.

To detect infected cells at different time points after FV infection, we performed flow cytometry after staining with antibodies allowing identification of the major cell subsets such as B cells, T cells, myeloid cells, and erythroblasts. For data visualization, we subjected the flow cytometry data of mWasabi^+^ cells to t-stochastic neighbor embedding (t-SNE), an unsupervised data analysis tool (16). The cells from different organs on each day of analysis were identified on the t-SNE plots by overlays (Fig. 2A), and this visualization revealed three phases of infection: the distribution changes from the early acute time point of day 2 postinfection (p.i.), when the initial virus load is still low (Fig. 1B), to the acute phase represented by days 4 and 7 p.i., which is characterized by a rapid increase in viral loads, and changes again in the late phase of infection as observed on days 14, 31, and 42 p.i., corresponding to the phase of stable, low viral loads. To identify the target cell populations infected at the different time points, we overlaid the t-SNE plot with the erythroblast marker Ter119, the B cell markers CD19 and B220, the myeloid cell markers CD11b, CD11c, and Gr1, or the T cell marker CD3 (Fig. 2B). On day 2 p.i., we found two clearly separated populations of infected cells in the t-SNE plots, which were constituted on the one hand by Ter119^+^ cells, i.e., erythroblasts, and cells that express B220, CD19, CD11b, and CD11c on the other hand, implying that this population comprised mainly myeloid cells as well as B cells which were not clearly separated by the t-SNE algorithm. In the acute phase of infection on days 4 and 7, we found a different distribution of infected cells in bone marrow and spleens compared to the early phase, as erythroblasts and a myeloid cell population positive for CD11b and Gr1 comprised a large proportion of infected cells. The infected cells in lymph nodes in the acute phase were still mainly comprised of Gr1^−^ myeloid cells and B cells, as observed in the early phase. In the late phase of infection, the infected cells in all organs were composed of three main populations as visualized by t-SNE: Gr1^−^ myeloid cells, B cells, and T cells.

To allow for quantification of the flow cytometry data, we performed a manual analysis that was informed by the prior t-SNE analysis (Fig. 2C). Therefore, we gated on Ter119^+^ erythroblasts (ery), Gr1^−^ CD11b^+^ CD11c^+^ myeloid cells (Gr1^−^ My), Gr1^+^ CD11b^+^ myeloid cells (Gr1^+^ My), CD19^+^ B220^+^ B cells, and CD4^+^ or CD8^+^ CD3^+^ T cells. Quantitative analysis of the number of infected cells in 10^6^ analyzed cells for the different subsets (Fig. 2C) as well as the frequency of these subsets within the total entity of detected FV-mWasabi-infected cells (Fig. 2D) confirmed the results of the nonsupervised t-SNE analysis. The dominant subset within the infected cell population in the early acute phase were Gr1^−^ myeloid cells in all organs (exhibiting significantly higher levels than at least three other subsets; *P < *0.05), followed by erythroblasts in bone marrow and spleen (Fig. 2C and D). Interestingly, the number of infected Gr1^−^ myeloid cells remained rather stable throughout the course of infection. Infected B cells were also detected in many mice at this early stage. In the acute phase of infection, on the other hand, a large proportion of FV-mWasabi-infected cells in bone marrow and spleen cells were erythroblasts, ranging in frequency from 30% to 80% of all infected cells (Fig. 2D). The frequency of FV-mWasabi-infected Gr1^+^ myeloid cells also increased in this phase of the infection, and importantly, we saw an increasing frequency of infected B cells of up to 20% and low frequencies of infected CD4^+^ T cells. While infected CD8^+^ T cells were detected from this phase of the infection in some samples, their number was very low. In the late phase of infection, the infected cells in all organs were dominated by myeloid cells, as in the early acute phase; however, a lower frequency of infected erythroblasts was detected in most mice, and especially in bone marrow, there was a notable contribution of Gr1^+^ myeloid cells to the infected cell population. While some mice showed a wider distribution of infected cell types at this stage of the infection, some mice harbored virus in only one or two cell types, while no virus was detected in the other cell types (Fig. 2C and D). Importantly, in both lymph nodes and spleen, but far less so in bone marrow, we saw FV-mWasabi-infected B cells and CD4^+^ T cells in half of the lymph node samples and in almost all spleen samples in the late phase of infection, which together constituted more than 20% of the infected cells in some of those mice.

### Myeloid cell subpopulations infected by FV-mWasabi.

As the myeloid cell populations carried the bulk of the virus load in the early phase and also in the late phase of FV infection, which is in contrast to previous reports (11), we aimed to characterize the FV-mWasabi-infected myeloid cells in more detail and subjected cells to staining with a myeloid cell-specific antibody panel. In the target cell staining we saw before that many cells stained positive for CD11b and CD11c; however, both markers do not allow for a clear discrimination of macrophages (MΦs) and dendritic cells. We therefore included the MΦ-specific cell marker CD68 (17) in the staining, and manual gating showed that almost 98% of FV-mWasabi-infected myeloid cells were highly positive for CD68, indicating that the vast majority of FV-mWasabi-infected CD11b^+^ cells were indeed MΦs and that only a small fraction of CD11b^+^ CD11c^+^ cells observed in the target cell analysis were dendritic cells.

**FIG 3:**
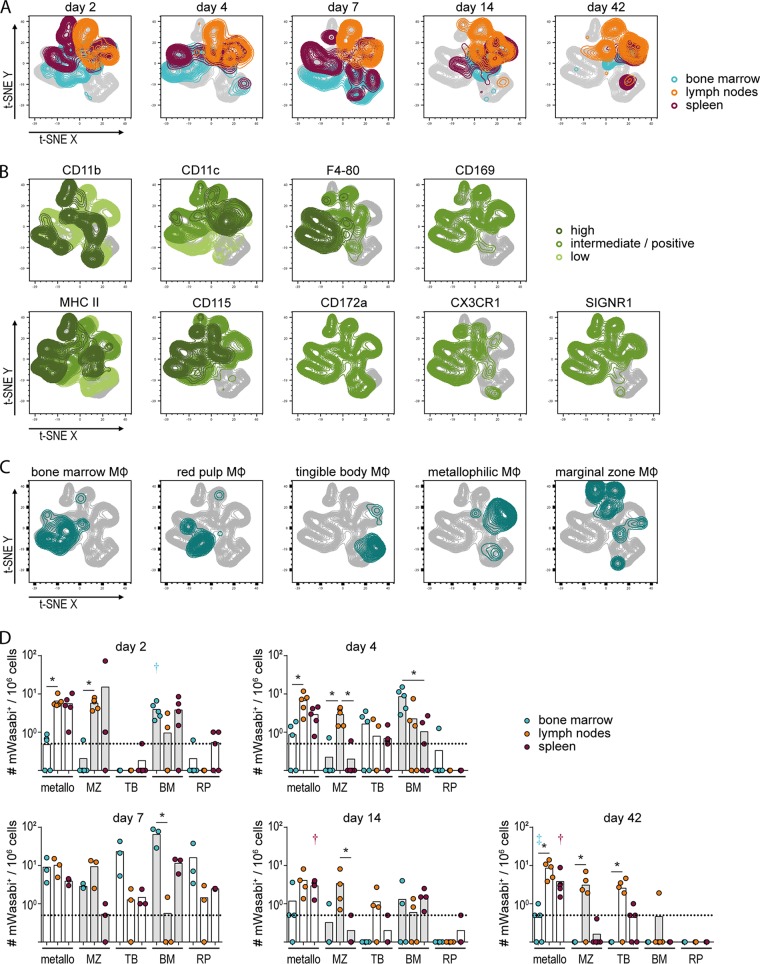
Analysis of FV-mWasabi-infected myeloid cells. C57BL/6 mice were infected with 20,000 SFFU FV-mWasabi, and infected cells were analyzed in bone marrow, lymph nodes, and spleens on days 2, 4, 7, 14, and 42 after FV-mWasabi infection. Cells were subjected to staining with a myeloid cell-specific antibody panel, and mWasabi^+^ CD68^+^ cells were subjected to a t-SNE analysis. (A) Manual gates on samples from individual organs and days of analysis were overlaid on the t-SNE plot to visualize the samples, and manual gates on individual cell markers (B) or manual gates on specific MΦ subtypes (C) were overlaid on the t-SNE plot to identify cell types. (D) Manual gating was performed to determine the number of mWasabi^+^ cells in metallophilic MΦs (metallo), marginal zone MΦs (MZ), tingible body MΦs (TB), bone marrow MΦs (BM), and red pulp MΦs (RP). Each circle represents the value for an individual mouse, and bars indicate mean values for groups of mice. The dotted lines indicate the detection limit. The data for each time point were obtained from one independent experiment per time point with *n* = 5 (day 2), 5 (day 4), 3 (day 7), 4 (day 14), or 5 (day 42). Statistically significant differences (*P < *0.05) between the numbers of infected cells of the different organs within a cell subset are indicated by lines and asterisks; color-coded dagger or double-dagger symbols indicate dominant subsets with significantly higher numbers of FV-mWasabi-infected cells compared to two (dagger) or three (double dagger) other subsets within one organ.

We therefore subjected FV-mWasabi^+^ CD68^+^ cells to a t-SNE analysis, which revealed a rather stable FV-mWasabi-infected MΦ population in lymph nodes, whereas the MΦ population in FV-mWasabi-infected spleens and bone marrow changed over the course of infection (Fig. 3A). We performed minimal manual gating to identify single MΦ-specific markers (Fig. 3B), and we also overlaid the t-SNE plots with manually gated specific MΦ populations (Fig. 3C). In the early acute and acute phases of FV infection, the majority of infected MΦs in bone marrow were identified as bone marrow MΦs (CD68^+^ CD11b^low^ F4-80^high^ MHC II^int^ CD115^int^ CD169^+^); in the late phase of infection, the population of MΦs in bone marrow contracted and was comprised of only low numbers of bone marrow MΦs and metallophilic MΦs (CD68^+^ CD11b^+^ F4-80^−^ CD169^+^). In spleens, the infected MΦ population changed over the course of infection, with red pulp MΦs (CD68^+^ CD11b^low^ F4-80^high^ MHC II^low^ CD115^+^ CD172a^+^), marginal zone MΦs (CD68^+^ CD11b^+^ F4-80^−^ SIGNR1^+^), tingible body MΦs (CD68^+^ CD11b^−^), and metallophilic MΦs being infected in the early acute and acute phase of infection, whereas metallophilic MΦs constituted the majority of infected spleen MΦs in the late phase of infection. The FV-mWasabi-infected MΦ population in lymph nodes remained rather stable throughout the whole course of infection and consisted mainly of marginal zone MΦs and metallophilic MΦs.

Manual gating of the flow cytometry data was performed as well for a quantitative analysis (Fig. 3D) and confirmed the results of the t-SNE analysis. Importantly, we confirmed that metallophilic MΦs, which have been demonstrated in other infection models to be important mediators for the initiation of adaptive immune responses (18), carried virus in all organs throughout the course of infection and may indeed be important for the orchestration of the FV-specific immune response.

### B cell subpopulations infected by FV-mWasabi.

Our target cell analysis revealed that the late phase of FV infection was characterized by the presence of FV-mWasabi-infected B cells, predominantly in lymph nodes and spleen. While the frequency of infected B cells in bone marrow was comparable to the frequency in lymph nodes and spleen cells in the acute phase, it was greatly reduced in the late phase of infection (Fig. 2C and D). As the FV-mWasabi-infected B cells were largely cleared from bone marrow but not from lymph nodes and spleen in the late phase of infection, we reasoned that the distinct localization of B cells in lymph nodes and spleen, or distinct phenotypes, might contribute to the maintenance of infected B cells in these organs. Therefore, we subjected lymph node cells and spleen cells from different time points after infection to staining with a B cell-specific antibody panel.

**FIG 4:**
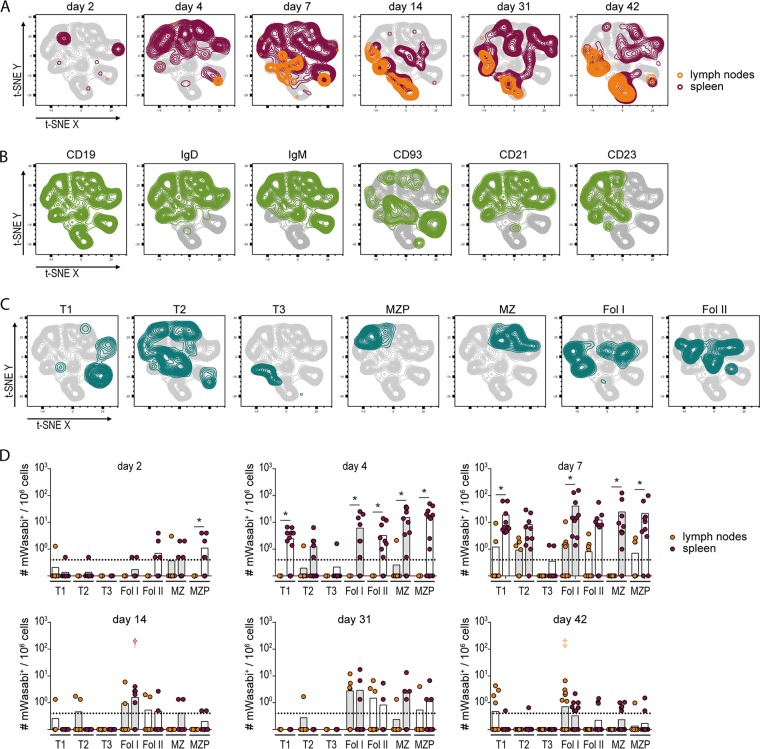
Analysis of FV-mWasabi-infected B cells. C57BL/6 mice were infected with 20,000 SFFU FV-mWasabi, and infected cells were analyzed in lymph nodes and spleens on days 2, 4, 7, 14, 31, and 42 after FV-mWasabi infection. Cells were subjected to staining with a B cell-specific antibody panel, and mWasabi^+^ CD3^−^ CD11b^−^ B220^+^ cells were subjected to a t-SNE analysis. (A) Manual gates on samples from individual organs and days of analysis were overlaid on the t-SNE plot to visualize the samples, and manual gates on individual cell markers (B) or manual gates on specific B cell types (C) were overlaid on the t-SNE plot to identify cell types. (D) Manual gating was performed to determine the numbers of transitory 1 (T1), T2, or T3 B cells, follicular B cell type I (Fol I) and II (Fol II), marginal zone B cells (MZ), and marginal zone precursor B cells (MZP). Each circle represents the value for an individual mouse, and bars indicate mean values for groups of mice. The dotted lines indicate the detection limit. The data for each time point were obtained from two (day 14 and day 31), three (day 2, day 4, and day 7), or six (day 42) independent experiments with *n* = 11 (day 2), 13 (day 4), 10 (day 7), 8 (day 14), 9 (day 31), or 26 (day 42). Statistically significant differences (*P < *0.05) between the numbers of infected cells of the different organs within a cell subset are indicated by lines and asterisks; color-coded dagger and double-dagger symbols indicate dominant subsets with significantly higher numbers of FV-mWasabi-infected cells compared to three (dagger) or four (double dagger) other subsets within one organ.

For visualization of the B cell flow cytometry data, we concatenated data of mWasabi^+^ cells from lymph nodes and spleens from all analyzed samples, gated on CD3^−^ CD11b^−^ B220^+^ cells and subjected the cells to a t-SNE analysis. Interestingly, identification of the lymph node and spleen samples from different time points after infection revealed that the infected B cell populations changed over the course of infection, similar to the infected cell population in general, with cells clustering differently in the acute phase than in the late phase of FV infection (Fig. 4A). Gating on individual B cell markers revealed that all analyzed cells stained positive for CD19, most cells stained for IgD and IgM, whereas CD93, CD21, and CD23 revealed distinct patterns (Fig. 4B). To facilitate identification of the B cell populations, we applied the characterization suggested by Allman and Pillai (19) and manually gated on transitory 1 (T1), T2, and T3 B cells, marginal zone precursor B cells (MZP), and marginal zone B cells (MZ), as well as on follicular B cell type I (Fol I) and type II (Fol II) (Fig. 4C). This analysis revealed that the infected B cell population of the acute phase comprised most of the developing B cell types, with a clearly different distribution of spleen- and lymph node-derived B cells. In the late phase of infection, however, spleen and lymph node cells largely clustered together, and the infected cells could be mainly identified as follicular B cells and MZ B cells. Interestingly, in the late phase of infection, both spleen and lymph nodes comprised FV-mWasabi-infected cells that were not overlaid by any of the cell subsets and were indeed negative for IgD and IgM (population in the lower middle of the t-SNE plot in Fig. 4A); furthermore, as these cells were CD19^+^ CD93^−^, we suggest that these cells were mature B cells that have undergone immunoglobulin class switching.

A manual gating was applied for quantification (Fig. 4D); it should be noted that at all time points, we found a proportion of mice where no FV-mWasabi-infected B cells of any subtype were detected, which is well in line with our findings in the target cell staining described above (Fig. 2C and D). The quantitative analysis revealed that during the acute phase of FV infection, spleens carried more than 10 times the number of infected B cells than lymph nodes, and the virus was equally distributed between T1, T2, Fol I, Fol II, MZP, and MZ B cells in the spleen. In the late phase of infection, on the other hand, we found similar frequencies of infected B cells in spleens and lymph nodes, and the infected B cell population was comprised mainly of Fol I and Fol II in both organs, and in addition of MZ and MZP B cells in spleens; Fol I B cells dominated the FV-mWasabi-infected B cell subsets in lymph nodes (exhibiting significantly higher levels than four other subsets; *P < *0.05). This is a very interesting finding, as follicular B cells, especially Fol I, reside in an immune-privileged site that is closed off to cytotoxic CD8^+^ T cells and therefore constitute a protected niche for FV.

### T cell subpopulations infected by FV-mWasabi.

**FIG 5:**
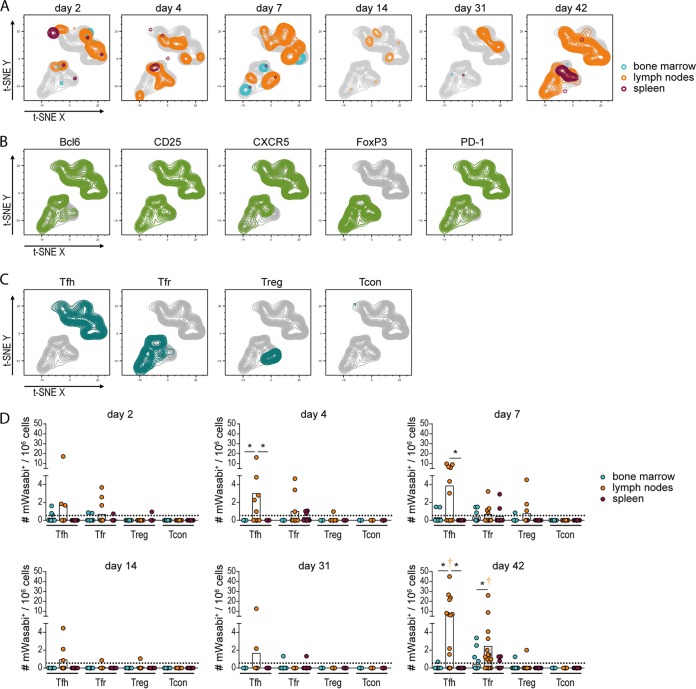
Analysis of FV-mWasabi-infected CD4^+^ T cells. C57BL/6 mice were infected with 20,000 SFFU FV-mWasabi, and infected cells were analyzed in bone marrow, lymph nodes, and spleens on days 2, 4, 7, 14, 31, and 42 after FV-mWasabi infection. Cells were subjected to staining with a CD4^+^ T cell-specific antibody panel, and mWasabi^+^ CD3^+^ CD4^+^ cells were subjected to a t-SNE analysis. (A) Manual gates on samples from individual organs and days of analysis were overlaid on the t-SNE plot to visualize the samples, and manual gates on individual cell markers (B) or manual gates on specific CD4^+^ T cell types (C) were overlaid on the t-SNE plot to identify cell types. (D) Manual gating was performed to determine the number of follicular helper T cells (Tfh), follicular regulatory T cells (Tfr), regulatory T cells (Treg), or conventional, nonfollicular nonregulatory CD4^+^ T cells (Tcon). Each circle represents the value for an individual mouse, and bars indicate mean values of groups of mice. The dotted lines indicate the detection limit. The data for each time point were obtained from two (day 4, day 14, and day 31), three (day 2 and day 7), or four (day 42) independent experiments with *n* = 12 (day 2), 9 (day 4), 10 (day 7), 8 (day 14), 9 (day 31), or 21 (day 42). Statistically significant differences (*P < *0.05) between the numbers of infected cells of the different organs within a cell subset are indicated by lines and asterisks; color-coded dagger symbols indicate dominant subsets with significantly higher numbers of FV-mWasabi-infected cells compared to two other subsets within one organ.

Another interesting cell subset that we observed in the FV-mWasabi-infected mice throughout the course of infection albeit at low levels was CD4^+^ T cells. To characterize the infected CD4^+^ T cells, we performed a CD4^+^ T cell subtype-specific staining of cells collected at different time points after FV-mWasabi infection and subjected mWasabi^+^ CD3^+^ CD4^+^ cells to a t-SNE analysis. The infected CD4^+^ T cells were found mainly in lymph node samples, but there was no apparent change in pattern over the course of infection (Fig. 5A), indicating that there was little change in cell subtypes constituting the infected CD4^+^ T cell population.

We performed minimal manual gating to identify individual cell markers on the t-SNE plot (Fig. 5B) and overlaid the t-SNE plots with manually gated CD4^+^ T cell subsets (Fig. 5C). The CD4^+^ cells clustered into two clearly separated populations in the t-SNE plot, and the clear separation of the two cell clusters could be attributed to the cells’ differential expression of FoxP3 (Fig. 5B). All cells were mostly positive for the other markers Bcl6, CD25, CXCR5, and PD-1 and could be identified as follicular helper T cells (Tfh) (CD3^+^ CD4^+^ CXCR5^+^ Bcl6^+^ FoxP3^−^ PD-1^high^), follicular regulatory T cells (Tfr) (CD3^+^ CD4^+^ CXCR5^+^ Bcl6^+^ FoxP3^+^ PD-1^high^), and regulatory T cells (Treg) (CD3^+^ CD4^+^ CXCR5^−^ CD25^+^ FoxP3^+^), respectively (Fig. 5C). Only a negligible contribution of nonfollicular, nonregulatory, conventional T cells (Tcon) (CD3^+^ CD4^+^ CXCR5^−^ FoxP3^−^) was recognized. The quantitative analysis showed that infected CD4^+^ T cells were detected in only some of the FV-mWasabi-infected mice, which corresponds with the findings from the target cell analysis described above (Fig. 2C and D), but they already appeared in early acute infection and increased in frequency toward the late phase when the infected CD4^+^ T cell population was clearly dominated by lymph node Tfh and Tfr (exhibiting significantly higher levels than two other subsets; *P < *0.05; Fig. 5D).

### Infection of follicular cells in the absence of effector T cell pressure.

The follicular B and T cells that are infected in the FV infection are residing in an immune-privileged niche, which may serve as a strategy for the virus to escape the immune response. We therefore wondered whether the cytotoxic CD8^+^ T cell response was responsible for driving the virus into this reservoir. To address this question, we performed infection experiments in highly FV-susceptible BALB/c mice. Because of their genetic background, BALB/c mice cannot mount an effective CD8^+^ T cell response against FV (MHC I haplotype H-2^d/d^ [20]) and mount inferior antibody responses (Rfv-3^s/s^, resistance factor encoding Apobec3 [21, 22]). In contrast to C57BL/6 mice, BALB/c mice develop severe splenomegaly and erythroleukemia after FV infection and show no control over viral loads.

**FIG 6:**
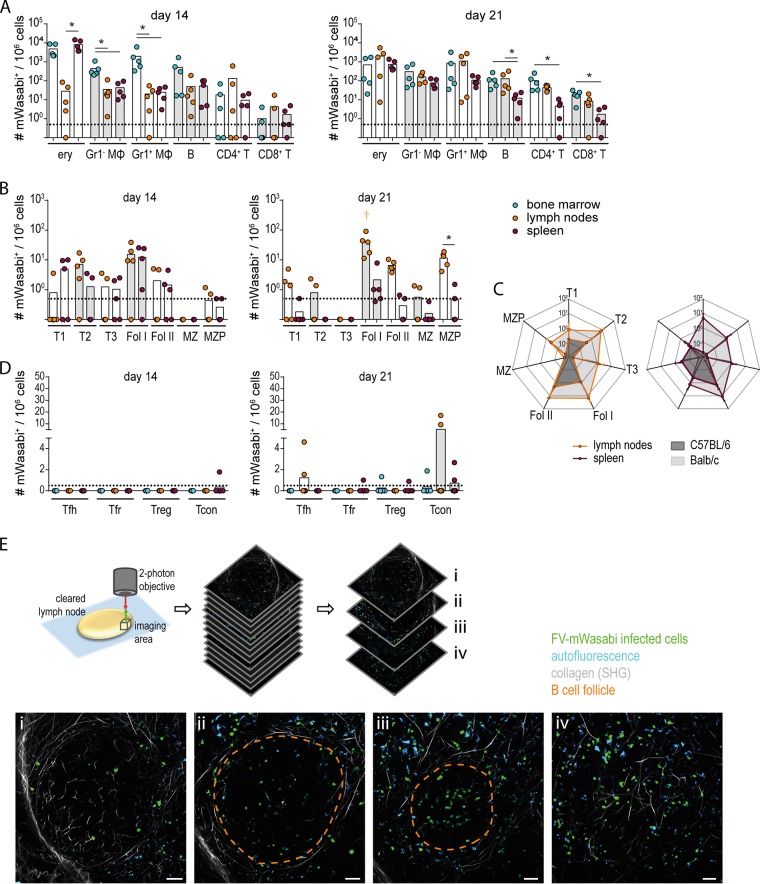
Analysis of the infected cell population in highly FV-susceptible BALB/c mice. BALB/c mice were infected with 500 SFFU FV-mWasabi, and cells from bone marrow, lymph nodes, and spleens were isolated on day 14 or 21 after FV-mWasabi infection. Cells were subjected to staining with a target cell-specific antibody panel (A), a B cell-specific antibody panel (B), or a CD4^+^ T cell-specific antibody panel (D) and analyzed by flow cytometry. Each circle represents the value for an individual mouse, and bars indicate mean values for groups of mice. The dotted lines indicate the detection limit. The data for each time point were obtained in one experiment per time point using five mice per time point. Statistically significant differences (*P < *0.05) between the numbers of infected cells of the different organs within a cell subset are indicated by lines and asterisks; color-coded dagger symbols indicate dominant subsets with significantly higher numbers of FV-mWasabi-infected cells compared to three other subsets within one organ. (C) Data from the target cell staining from C57BL/6 mice as shown in Fig. 2 and from BALB/c mice are displayed in radar charts for direct comparison. Dots indicate mean values, and whiskers indicate standard deviations. (E) Twenty-one days after infection, the lymph nodes from BALB/c mice were obtained and subjected to CUBIC clearing, followed by two-photon microscopy. Images from a 3D space of 590 µm by 590 µm by 150 µm were acquired (see Movie S1 for a sequence of all images). mWasabi^+^ cells could be clearly detected in the area surrounding the B cell follicle (i and iv) as well as within the B cell follicle (ii and iii). The scale bars represent 50 µm. The broken orange line (ii and iii) indicates the contour of the B cell follicle identified by the visualization of collagen structures by the second harmonic generation signal.

When we analyzed the infected cell population in BALB/c mice 14 and 21 days after infection (Fig. 6A), the distribution of infected cell populations was similar to the target cell distribution in C57BL/6 mice in the acute phase of infection: a broad range of cells was infected by FV-mWasabi, and the frequency of infected cells tended to be highest in erythroblasts, followed by Gr1^−^ and Gr1^+^ myeloid cells and B cells. Interestingly, we found a substantial contribution of CD4^+^ T cells and CD8^+^ T cells to the infected cell population in almost all mice by day 21, whereas we had observed these cells more sporadically among the FV-mWasabi-infected cells in C57BL/6 mice.

10.1128/mBio.00004-19.1 MOVIE S1 Twenty-one days after infection, lymph nodes from BALB/c mice were obtained and subjected to CUBIC clearing, followed by two-photon microscopy. Images from a 3D space of 590 µm by 590 µm by 150 µm were obtained, acquiring an image every 1.5 µm, and combined in a z-stack. A 3D surface rendering was integrated in the movie to outline the B cell follicle that was identified by collagen structures visualized by the second harmonic generation signal. Download Movie S1, MPG file, 43.8 MB. Copyright © 2019 Windmann et al. 2019 Windmann et al. This content is distributed under the terms of the Creative Commons Attribution 4.0 International license.

When we analyzed the FV-mWasabi-infected B cells (Fig. 6B and C), the distribution was slightly but not significantly different from that observed in C57BL/6 mice, as more infected B cells tended to exhibit a transitory phenotype than in C57BL/6 mice at 14 days p.i. and fewer MZ and MZP B cells were found. Importantly, we found a high frequency of infected Fol I and Fol II B cells in lymph node samples from most mice on day 14 and in all lymph node samples on day 21, with Fol I B cells being the dominant FV-mWasabi-infected B cell population in lymph nodes at this time point (exhibiting significantly higher levels than four other subsets; *P < *0.05) (Fig. 6B). The frequency of infected follicular B cells in the spleen was relatively low, but it has to be taken into consideration that at the analyzed time points, mice have developed severe splenomegaly due to massive erythroblast proliferation, leading to a relatively reduced frequency of B cells in the spleen cell population, and the spleens have also largely lost their normal structure (data not shown). When we analyzed the frequency of specific CD4^+^ T cell subsets among the infected cells, we did not see a major contribution of the follicular CD4^+^ T cells, although some mice harbored FV-infected Tfh and Tfr at very low frequency (Fig. 6D). Interestingly, some mice harbored a higher proportion of infected Tcon, which we had not found in C57BL/6 mice.

To directly visualize FV-mWasabi-infected cells in B cell follicles, we removed lymph nodes from BALB/c mice 21 days after infection, fixed and cleared the lymph nodes using the CUBIC protocol (23), and subjected them to two-photon microscopy. Scanning different areas of the lymph nodes, we could clearly detect FV-mWasabi-infected cells in the area around the B cell follicle as well as in large numbers within the B cell follicle, confirming the results obtained by flow cytometry (Fig. 6E; see also Movie S1 in the supplemental material). Interestingly, some of the infected cells within the follicle seemed to cluster, suggesting that infection of by-standing cells may occur in this location.

Overall, the high level of infected follicular B cells in the FV-mWasabi infection of highly susceptible BALB/c mice suggests that infection of follicular cells occurs in the absence of cytotoxic T cell pressure and irrespective of peripheral control.

**FIG 7:**
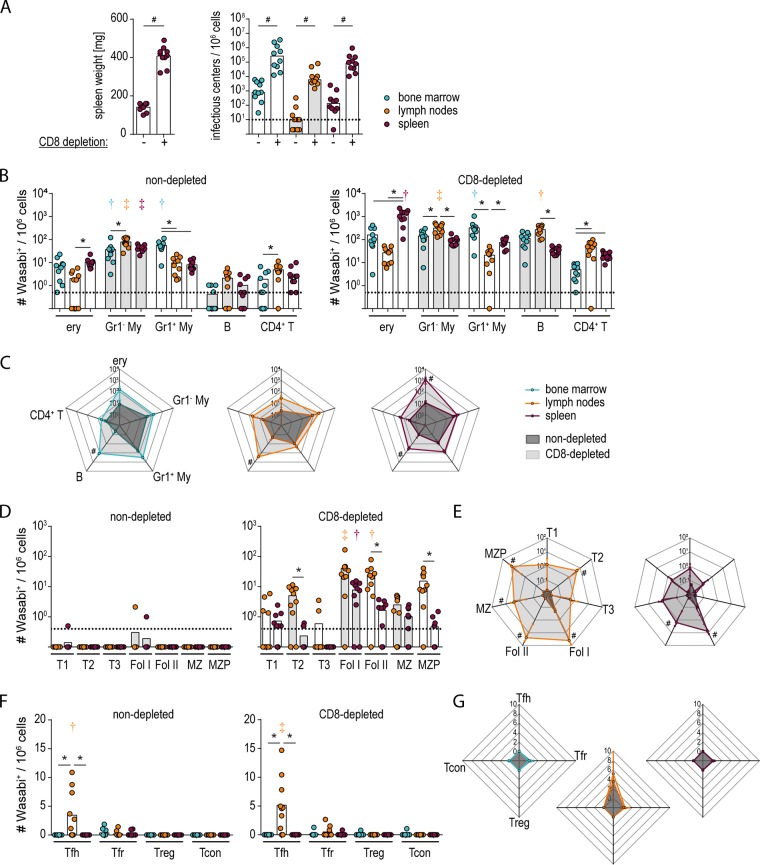
Analysis of the infected cell population in C57BL/6 mice depleted of CD8^+^ T cells. C57BL/6 mice were infected with 20,000 SFFU FV-mWasabi, and depleted of CD8^+^ T cells from day 0 to day 13. Mice were sacrificed 14 days after FV-mWasabi infection, and spleen weights (A, left panel) and viral loads in bone marrow, lymph nodes, and spleens were analyzed (A, right panel). For identification of infected cell populations, cells were subjected to staining with a target cell antibody panel (B and C), a B cell-specific antibody panel (D and E), or a CD4^+^ T cell-specific antibody panel (F and G) and analyzed by flow cytometry. (A, B, D, and F) Each circle represents the value for an individual mouse, and bars indicate mean values for the groups of mice. The dotted lines indicate the detection limit. (C, E, and G) Data points on the radar charts indicate mean values. The standard deviations are indicated by whiskers. The data were obtained from one experiment using 10 mice per group. Statistically significant differences (*P < *0.05) between nondepleted and CD8-depleted mice are indicated by number or pound symbols. Significant differences between the numbers of infected cells of the different organs within a cell subset are indicated by lines and asterisks. Color-coded dagger or double-dagger symbols indicate dominant subsets with significantly higher numbers of FV-mWasabi-infected cells compared to two (dagger) or three (double dagger) other subsets within one organ (B and F) or compared to three (dagger) or four (double dagger) other subsets within one organ (D).

To obtain a more definitive idea about the influence of CD8^+^ T cell pressure on the infected cell population, we infected C57BL/6 mice with FV-mWasabi, depleted mice of CD8^+^ cells starting on the day of infection, and analyzed the infected cells at day 14, i.e., in the late phase of infection when the target cell distribution does not change anymore. As expected, the depletion of CD8^+^ T cells led to an impaired control of the FV-mWasabi infection, and mice exhibited increased spleen weight and increased viral loads in all organs compared to FV-mWasabi-infected, nondepleted mice (Fig. 7A). When we analyzed the FV-mWasabi-infected cells by target cell staining, we found that while myeloid cells were dominant subsets in both nondepleted and CD8^+^ T cell-depleted mice, erythroblasts in spleens and B cells in lymph nodes also dominated the FV-mWasabi-infected cell population in CD8^+^ T cell-depleted mice (Fig. 7B). Overall, the numbers of infected cells tended to increase in all subsets in CD8^+^ T cell-depleted mice compared to nondepleted mice, with significantly higher numbers of FV-mWasabi-infected B cells in all organs and of infected erythroblasts in spleens (Fig. 7B and C). While almost all B cell subsets showed higher numbers of FV-mWasabi-infected cells in CD8^+^ T cell-depleted mice, the infected B cell population in depleted mice, especially in lymph nodes, was clearly dominated by follicular B cells (exhibiting significantly higher levels than at least three other subsets; *P < *0.05; Fig. 7D and E). The infected CD4^+^ T cell population, on the other hand, did not show any significant differences between nondepleted and CD8^+^ T cell-depleted mice and was dominated by Tfh cells in both groups (exhibiting significantly higher levels than at least two other subsets; *P < *0.05) (Fig. 7F and G).

Taken together, the data from both the highly susceptible BALB/c mice and the CD8^+^ cell-depleted C57BL/6 mice that exhibit impaired control of FV infection clearly show that infection of follicular B and T cells occurs in the absence of CD8^+^ T cell pressure, and indeed reaches higher levels in these cells compared to nondepleted C57BL/6 mice. These results suggest that the extent of infection of follicular B cells mirrors the overall viral loads during acute infection, but under normal immune pressure during an ongoing infection, lymph node follicles become a privileged hiding place for the virus.

## DISCUSSION

Our data show that the establishment of reservoirs in the B cell follicle as an immunologically privileged niche is a property not only of HIV and SIV but also of the murine Friend retrovirus. Because of distinct target cell tropism, the infected follicular cell types differ for the viruses, whereas the effect is the same, as the privileged hiding place, be it follicular T cells (1–3) or follicular B cells, allows the viruses to evade the immune response.

In the past, some studies have addressed the characterization of infected cells in FV infection, using either FV-specific antibody staining (24–26) or an infectious center assay of sorted spleen cells (11) to identify FV-harboring cells. Interestingly, our observations in the acute phase of infection agree with these reports, as their experiments also demonstrated an infected target cell pool that was dominated by erythroid cells, followed by B cells and myeloid cells. Hasenkrug et al. analyzed FV-producing cells in the chronic phase of FV infection, and they showed that the productively infected cell population was heavily dominated by B cells, representing ∼90% of virus-releasing cells in chronically infected mice (11). Our analyses, on the other hand, showed that the majority of infected cells in the late phase of infection were myeloid cells in most mice; the discrepancy may be explained by constraints of the available techniques at the time, especially as virus loads tend to be very low at this stage. Of course, it also has to be noted that our analysis, as well as analyses detecting FV-infected cells by antibody staining, reveals FV-infected cells, which do not necessarily actively produce virus, in contrast to the analysis of virus-producing cells presented before. In this context, it also should be noted that our study harbors some limitations, as we are investigating FV infection but detecting only F-MuLV by its fluorescent label. It would certainly be of great interest to see whether the dynamics of F-MuLV and SFFV differ, which would require a recombinant, differentially labeled SFFV.

MΦs but also B cells are known to express retroviral restriction factors such as Apobec proteins or SAMHD1, which can interfere with the production of infectious retrovirus progeny (27–29). When SAMHD1-deficient mice were infected with FV in the past, no effect on total virus loads was observed (29); unfortunately, the effect on distinct cell types was not investigated, and it would be intriguing to analyze whether deficiency in SAMHD1 would lead to changes in the composition of the infected cell population. Furthermore, the resistant C57BL/6 mice that were used here in most experiments are notorious for expressing an active variant of Apobec3 (Rfv3^r^), which increases the virus mutation rate, thereby increasing the production of noninfectious virus particles and leading to a superior antibody response (30). It has been suggested before that B cell infection plays an important role in the induction of FV-specific immune responses, as FV-infected B cells are potent stimulators of CD8^+^ T cells (31). As we saw early infection of B cells, our results suggest that B cells may contribute as antigen-presenting cells to the induction of FV-specific immune responses *in vivo*. Furthermore, the contribution of B cells to the early infected cell population and the appearance of infected B cells with developing phenotypes suggest that differentiating B cells, possibly with FV specificity, may carry the virus along into B cell follicles. How much the infection of follicular cells contributes to the spread of the virus can only be extrapolated from the data we present here. While it has been shown before that direct, CD40/CD154-dependent interactions of CD4^+^ T cells and B cells in germinal centers contribute to virus spread and pathogenesis in the mouse LP-BM5 leukemia virus model (32, 33), similar to CD40-dependent interactions of CD4^+^ T cells and dendritic cells in HIV infection (34), we did not observe an increase in Tfh infection despite the elevated levels of infected follicular B cells in BALB/c mice or in CD8^+^ T cell-depleted C57BL/6 mice, arguing against direct spread of FV-mWasabi from infected B cells to Tfh cells in the B cell follicle as an important infection-promoting mechanism.

We could show that erythroid cells become infected at significant numbers very early in FV infection, which is probably due to their high proliferative activity and also to a high level of expression of the FV receptor molecule mCat-1 (35), making these cells attractive targets for FV. The frequency of infected erythroid cells drops to a comparably low level in the late phase of the infection, as infected erythroblasts are efficiently eliminated by cytotoxic CD8^+^ T cells (36). The high frequency of infected MΦs in the very early phase of infection and their constant number throughout the course of FV infection, on the other hand, are more difficult to explain. It is generally accepted that simple retroviruses infect only dividing cells, suggesting that the infection of MΦs might have occurred at the level of a proliferating precursor. On the other hand, *in vitro* experiments showed that MΦs at a certain state of activation allow infection even though they are not replicating (37). Furthermore, it can be speculated that this permissiveness may be associated with MΦ function: it has been shown in other virus infections that MΦs are often highly susceptible to infection and show increased permissiveness for virus replication compared to other cell types, in fact enhancing virus replication and load and thereby facilitating the induction and orchestration of an effective immune response (18).

As the MΦs outnumbered all other professional antigen-presenting cells in the FV-mWasabi-infected cell pool, their contribution to the induction of the FV-specific immune response is likely to be high. While the MΦs are not located in any immune-privileged sites, they have been shown to be protected from elimination by cytotoxic cells specific for different pathogens, including HIV (38), by mechanisms that may involve serpin serine protease inhibitors that interfere with cytotoxic molecules such as granzymes as demonstrated for dendritic cells (39). In the FV model, it was demonstrated before that FV-infected Gr1^+^ myeloid cells express large amounts of the inhibitory ligand PD-L1, thereby preventing their elimination by CD8^+^ T cells (36). In the FV model as well as in other retroviral infections, it has also been shown that myeloid-derived suppressor cells expand upon infection and dampen adaptive immune responses (40–44). However, whether or not these suppressor cells were infected by the virus was not addressed. A clear distinction of myeloid cell subsets by surface markers can be difficult to achieve and functional assays may be more informative; therefore, it would be interesting to analyze whether some of the FV-mWasabi-infected myeloid cells may in fact exhibit immunosuppressive functions.

The roles that the different cell subsets that are infected with FV play in the induction and promotion of the FV-specific immune response, and in the propagation of the virus and the maintenance of a persistent infection, shall be addressed in the future. Of particular interest is the infected cell distribution following therapeutic interventions. Previous studies showed that the therapeutic depletion of Tregs results in increased effector functions of CD8^+^ T cells and a reduction in chronic viral loads (8), and therapeutic efficacy was further improved when inhibitory ligands were blocked (9), but these therapeutic interventions were not sufficient to completely eliminate persistent FV. On the basis of the results of analysis of FV-infected cells shown here, we hypothesize that increased effector functions of CD8^+^ T cells could lead to elimination only of accessible cells, whereas follicular B and T cells would escape this control. Licensing of CD8^+^ T cells or a manipulation of the B cell follicle structure, as has been suggested for HIV therapy (45), may be feasible approaches for the eradication of the follicular reservoir and may complement immune therapeutic interventions described before.

Our results demonstrate that the infection of follicular cells is a shared property of HIV and the simple gammaretrovirus FV, making FV a valuable model not only for studying mechanisms of immune control but also for the development of therapeutic approaches targeting infected follicular cells, which are considered very important targets in HIV cure approaches.

## MATERIALS AND METHODS

### Cells.

A murine fibroblast cell line from Mus dunni (46) and the murine hybridoma cell line 720 (47) were maintained in RPMI medium supplemented with 10% heat-inactivated fetal bovine serum and 50 U/ml gentamicin. The human embryonic kidney cell line 293T was maintained in DMEM medium supplemented with 10% heat-inactivated fetal bovine serum, 50 µg/ml gentamicin, and 20 µg/ml ciprofloxacin (all cell culture reagents were obtained from Invitrogen/Gibco, ThermoFisher Scientific, Karlsruhe, Germany). Cells were maintained in a humidified 5% CO_2_ atmosphere at 37°C.

### Mice.

Female C57BL/6 and BALB/c mice were purchased from Envigo Laboratories (Rossdorf, Germany). Mice were between 7 to 10 weeks old at the onset of the experiments.

All mouse experiments were performed with permission from the authorities (Northrhine-Westphalia State Office for Nature, Environment and Consumer Protection, LANUV NRW) and in accordance with the national law and the institutional guidelines of the University Hospital Essen, Germany.

### Viruses.

A modified Friend murine leukemia virus was created that encodes the green fluorescent protein mWasabi (12) fused to the C terminus of the envelope open reading frame linked by the self-cleaving 2A peptide of porcine teschovirus (13) (Fig. 1A). Cloning was performed using plasmid pFB29 that encodes a permuted clone of F-MuLV strain FB29 (48) (pFB29 kindly provided by Marc Sitbon, Institut Génétique Moléculaire de Montpellier, Montpellier, France; kindly transferred by Masaaki Miyazawa, Kindai University Faculty of Medicine, Osaka, Japan). A ClaI-AscI fragment containing part of F-MuLV Env p15E, a glycine-serine linker, mWasabi, and F-MuLV U3 was synthesized (GeneArt, ThermoFisher, Regensburg, Germany) and subcloned into pBluescript; the 2A sequence was assembled from oligonucleotides (Biomers, Ulm, Germany) and inserted between the glycine-serine linker and the mWasabi coding sequence. The resulting ClaI-AscI fragment containing the C terminus of p15E, 2A peptide, mWasabi, and U3 was introduced into pFB29 with ClaI and AscI. For reconstitution of the mWasabi-encoding F-MuLV-mWasabi, the genome was released from the pFB29-2A-mWasabi plasmid by HindIII digestion, religated, and transfected into 293T cells. Recovered virus was purified from supernatants of transfected 293T cells and passaged on Mus dunni cells, and virus was purified by sucrose cushion ultracentrifugation.

For reconstitution into a complex with SFFV, we constructed an infectious molecular SFFV clone. For this, an LTR was synthesized and cloned into plasmid pMK (pMK-LTR; GeneArt, ThermoFisher, Regensburg, Germany). The permuted SFFV clone pBR322-SFFV (kindly provided by Leonard Evans, NIAID, NIH, Hamilton, MA) was linearized with AscI between the U3 and R sequences of the LTR and cloned into the AscI site of pMK-LTR. The pBR322 backbone was removed by digestion with HindIII and religation, resulting in the colinear clone pMK-SFFV.

To reconstitute the complex of F-MuLV-mWasabi and SFFV (FV-mWasabi), we transfected pMK-SFFV into Mus dunni cells and superinfected the cells with F-MuLV-mWasabi. The complex was passaged on Mus dunni cells, and virus stocks were prepared as described above. The presence of SFFV in the complex was verified by staining infected cells with a polyclonal rat anti-SFFV-gp55 antibody, followed by staining with a secondary APC-labeled antibody and detection by flow cytometry. The ratio of F-MuLV-mWasabi and SFFV was ∼1:2.

To obtain an *in vivo* stock of FV-mWasabi, BALB/c mice were infected with the purified complex; spleens were collected 15 days after infection when splenomegaly was established, and 15% spleen cell homogenates were prepared and stored at −80°C. The infectious titer of the stock, expressed as spleen focus-forming units (SFFU) per milliliter of virus stock, was determined by infection of CB6F1 mice and determination of spleen foci 10 days after infection using fixation by Bouine’s fixative as described before (49).

### FV infection.

C57BL/6 mice were infected with 20,000 SFFU of FV-mWasabi by intravenous injection, and BALB/c mice were infected with 500 SFFU of FV-mWasabi. For the analysis of infected cells, mice were sacrificed at different time points after infection, and spleens, lymph nodes, and bone marrow were collected.

### Analysis of viral loads.

Dilutions of single-cell suspensions of spleen, lymph node, and bone marrow cells were seeded onto Mus dunni fibroblasts and incubated for 3 days until cells reached confluence. Infected cells were detected by immunocytochemical staining using the hybridoma-derived antibody 720 as described before (14).

### Flow cytometry and data analysis.

Single-cell suspensions of spleen, lymph node, and bone marrow cells were stained with the following antibody panels (antibodies were obtained from BioLegend [San Diego, CA], unless indicated otherwise).

The target cell antibody panel consisted of phycoerythrin-labeled anti-mCat-1 (PE-α-mCat-1), PE-TexasRed-α-CD11c, PerCP-α-CD4, PE-Cy7-α-Ter119, APC-α-CD71, AlexaFluor680-α-CD19, APC-Cy7-α-CD3, BrilliantViolet421-α-Gr1, BrilliantViolet510-α-B220, BrilliantViolet605-α-CD11b, and BrilliantViolet650-α-CD8.

The B cell antibody panel (spleen, lymph nodes) consisted of PE-α-CD93, PE-TexasRed-α-IgM, PerCP-α-CD1a, PE-Cy7-α-CD23, APC-α-CD21, AlexaFluor680-α-CD19, APC-Cy7-α-CD3, BrilliantViolet421-α-CD5, BrilliantViolet510-α-B220, BrilliantViolet605-α-CD11b, and BrilliantViolet650-α-IgD.

The follicular T cell antibody panel consisted of PE-TexasRed-α-PD1, PerCP-α-CD25, PE-Cy7-α-CXCR5, APC-α-Bcl6, AlexaFluor680-α-CD3, APC-Cy7-α-CD4, BrilliantViolet421-α-FoxP3, BrilliantViolet510-α-CD44, BrilliantViolet605-α-CD11b, and BrilliantViolet650-α-CD69.

The macrophage antibody panel consisted of PE-TexasRed-α-CD172a, PerCP-α-CD169, PE-Cy7-α-CD115, APC-α-SIGNR1, AlexaFluor680-α-F4/80, APC-Cy7-α-CX3CR1, BrilliantViolet421-α-CD68, BrilliantViolet510-α-CD11c, BrilliantViolet605-α-MHC II, and BrilliantViolet650-α-CD11b.

Staining was performed in FACS buffer for 1 h at room temperature or overnight at 4°C. Fc receptor binding was blocked by incubation with rat-α-mouse CD16/CD32 (Becton, Dickinson, Mountain View, CA) for 15 min prior to antibody staining. The fixable viability dye Zombi UV was included in all staining to exclude dead cells from analysis. Intracellular staining was performed using a FoxP3/transcription factor staining buffer kit (Bcl6 and FoxP3; eBioscience, ThermoFisher Scientific, Darmstadt, Germany) or using 2% formaldehyde diluted in PBS for fixation and 0.1% saponin buffer for permeabilization and intracellular staining (CD68).

Data were acquired on a 4-laser, 15-color LSR II flow cytometer (Becton, Dickinson, Mountain View, CA). Data were analyzed using FlowJo software (version 10.3.0; FlowJo LLC, Ashton, OR), using the t-SNE FlowJo plug-in for t-stochastic neighbor embedding.

### CD8^+^ cell depletion.

For the analysis of the effect of CD8^+^ T cells on the target cell distribution, mice were depleted of CD8^+^ T cells by intraperitoneal injection of 250 µl of hybridoma supernatant containing the CD8-specific antibody 169.4 (50). Antibody was applied every other day, starting on the day of infection.

### Microscopy.

For the detection of infected cells by two-photon microscopy, infected mice were sacrificed by CO_2_ asphyxiation and were immediately perfused transcardially with 15 ml cold EDTA/PBS followed by 20 ml of cold 4% formaldehyde in PBS (pH 7.4). Axillary, brachial, and inguinal lymph nodes were removed and incubated with shaking at 4°C in 4% formaldehyde in PBS for 4.5 h. Samples were washed twice with PBS for at least 2 h and incubated with CUBIC-1 reagent described by Susaki et al. (23) for 5 days with gentle shaking at 37°C; CUBIC-1 reagent was renewed after 2 and 4 days of incubation. Optically cleared lymph nodes were analyzed by two-photon microscopy, using a Leica TCS SP8 MP microscope (Leica Microsystems, Mannheim, Germany) with HCX IRAPO L 25×/0.95-NA water immersion objective, two internal hybrid reflected-light detectors (HyD), and two external photomultiplier tubes (PMT). Imaging was performed with a titanium-sapphire laser (Coherent Cameleon Vision II, Santa Clara, CA, USA) tuned to 950 nm. FV-mWasabi-infected cells (HyD, 525/50 filter), autofluorescent structures (PMT, 585/40 filter; PMT, 650/50 filter) and collagen visualized by second-harmonic-generation (SHG) signal (HyD, 460/50 filter) were detected. Imaris 9.2.1 software (Bitplane AG, Zurich, Switzerland) was used for reconstruction of raw data.

### Statistical analysis.

Analysis of data for statistically significant differences was performed either using an unpaired *t* test for the comparison of parametric data from two groups or using a nonparametric one-way analysis of variance on ranks with Dunn’s multiple-comparison procedure in GraphPad Prism 5 software.
